# Inflammatory Responses to Zn/Cu-Containing Welding Fume in Human Alveolar Epithelial and Macrophage Cell Lines, with MIP-1β/CCL4 as a Much More Sensitive Macrophage Activation Marker than IL-8 and TNF-α

**DOI:** 10.3390/ijms26083843

**Published:** 2025-04-18

**Authors:** Jan Steffens, Katharina Kuth, Thomas Kraus, Wolfgang Dott, Sabrina Michael, Ralf Baumann

**Affiliations:** 1 Institute for Occupational, Social and Environmental Medicine, Medical Faculty, RWTH Aachen University, Pauwelsstrasse 30, 52074 Aachen, Germany; 2 Institute for Translational Medicine, Medical Faculty, Medical School Hamburg (MSH), Am Kaiserkai 1, 20457 Hamburg, Germany; 3 Institute of Hygiene and Environmental Medicine, Medical Faculty, RWTH Aachen University, Pauwelsstrasse 30, 52074 Aachen, Germany

**Keywords:** welding and metal fumes, zinc (Zn), copper (Cu), metal (nano)particle exposure, nanotoxicology, oxidative stress, inflammatory response, cytokines (IL-6, IL-8, MIP-1β/CCL4, TNF-α), early-stage marker, occupational health

## Abstract

Zinc (Zn)- and copper (Cu)-containing welding fumes elevate inflammatory markers (CRP, TNF-α, IL-6, IL-8) in healthy individuals and welders. Zn- and Cu-containing nanoparticles are toxic to human macrophages. Therefore, ZnO exposure limits are under discussion. In this study, the effects of Zn/Cu-containing welding fume suspensions on A549 alveolar epithelial cells (exposure concentrations: 0.01/0.1/1/10/100 µg/mL) and THP-1 macrophages (additionally 0.001 µg/mL) were investigated over a period of 48 h. Effects on apoptosis, cytotoxicity, genotoxicity, superoxide dismutase (SOD) activity, and cytokine levels (IL-6, IL-8, MIP-1β/CCL4, TNF-α) were evaluated. Welding fume exposure increased SOD activity, and it increased Annexin-V binding and cytotoxicity effects starting at 10 µg/mL in A549 cells and particularly in THP-1 macrophages. A549 cells showed increased IL-6 at 10 and 100 µg/mL, and significant IL-8 release occurred at 10 µg/mL for A549 and 0.1 µg/mL for macrophages. Exposed macrophages released TNF-α at 1 µg/mL after 24 and 48 h and MIP-1β/CCL4 at 0.01 µg/mL after 6 h and at 0.001 µg/mL after 48 h. No genotoxic effects were detected. MIP-1β/CCL4 is a sensitive new biomarker for human macrophages exposed to Zn/Cu-containing welding fumes. The findings suggest that Zn/Cu particles affect lung cells already at doses below current occupational thresholds.

## 1. Introduction

Welding, a versatile method used to join metallic components, is crucial in manufacturing. But it exposes workers to welding fumes containing a variety of gases and single or agglomerated particles with diameters ranging from 10 to 400 nm [1]. These fumes can be inhaled [2], leading to inflammatory responses in the respiratory system and systemic subclinical inflammatory effects [3,4]. Epidemiological studies have shown that welders have an increased risk of both systemic and pulmonary diseases, including cardiovascular diseases, occupational asthma, chronic obstructive pulmonary disease (COPD), chronic bronchitis, and pneumonia [3,4,5,6]. Welders experience more frequent, longer-lasting, and severe respiratory infections [3]. Exposure to fumes containing metals like nickel, chromium, manganese, and iron has been associated with increased soluble cytokines, such as IL-8, in post-shift nasal lavage fluid (NALF) [7]. Significant systemic increases of CRP and especially IL-6 were reported after short-term exposures to welding fumes [8]. Of note, IL-6 is a strong risk marker [9] and a mediator for future cardiovascular diseases [10].

In modern joining technology, especially in the automotive industry, an increasing number of welding fumes contain zinc (Zn) and copper (Cu) [11]. Controlled short-term Zn exposure caused increased levels of TNF-α, IL-6, and IL-8 in bronchoalveolar lavage (BAL) fluid of exposed welders [12] and increased levels of TNF-α and IL-8 in BAL of exposed healthy subjects [13]. Healthy individuals showed increased systemic IL-6 levels after ZnO exposure [14]. Moreover, we have shown that controlled short-term exposure of healthy individuals with a Zn/Cu-containing welding fume caused significantly increased levels of C-reactive protein (CRP) and serum amyloid A (SAA) in nasal mucosal lining fluid [15] that preceded and accompanied increased systemic levels of IL-6 at 10 h, and CRP and SAA at 29 h after exposure compared to unexposed control individuals [15,16,17,18]. Repeated controlled exposures to Zn- and Cu-containing welding fumes of healthy individuals for 6 h on 4 consecutive days showed a persistent systemic CRP increase, suggesting that long-term exposure to these metals may lead to prolonged subclinical inflammation [19].

Pollutants and metal fumes reach the exposed subject primarily via the airways. Alveolar macrophages are key players in lung inflammation and are involved in the defense against pathogenic microbes and inhaled pollutants. In a previous study, we have shown that the toxicological effects of different ambient (urban traffic or rural) PM10 samples containing different trace metals caused distinct dose-dependent toxicological responses in human alveolar epithelial cells (A549) and murine macrophages (RAW264.7) [20]. Furthermore, we have shown that exposure of THP-1 macrophages in vitro to Zn/Cu-containing particle suspensions leads to an increased expression of lncRNAs [21] and a reduced response to bacterial products [22]. In another in vitro study, Cu- and Zn-based nanoparticles were identified to be the most toxic among 24 tested different metal-based nanoparticles for A549 cells and THP-1 macrophages, based on the cytotoxicity MTT and Neutral Red assays [23]. Moreover, exposure of macrophages to ZnO nanoparticles decreased lysosomal stability and increased cell death [24]. In addition, Zn/Cu-particles have been found to negatively impact lung function in isolated perfused mouse lungs (IPLs) [25].

Several studies have investigated oxidative stress effects of welding fume and metal oxide (nano)particles, using the antioxidant enzyme superoxide dismutase (SOD) as an indicator. A study performed in Turkey on welders revealed a significant decrease in SOD activity compared to controls [26]. The effects of ZnO nanoparticles on male Wistar rats demonstrated a significant reduction in SOD activity and gene expression in the liver [27]. Additionally, macrophages exposed in vitro to CuO nanoparticles underwent cell death through the misfolding of Cu/Zn superoxide dismutase 1 (SOD1) [28].

To further investigate the cellular responses of A549 alveolar epithelial cells and PMA-differentiated THP-1 macrophages in vitro to a Zn/Cu-containing welding fume suspension in this study, we examined the concentration- and time-dependent effects on cell toxicity via viability, cell death through apoptosis, cellular stress as indicated by SOD activity levels, and immunoregulatory effects through selected secreted cytokines. The cytokines IL-6, TNF-α, and the chemokine IL-8 are central proinflammatory mediators and are also involved in metal fume fever [12]. IL-6 is a main mediator for the orchestration of the acute-phase reaction [29]. Levels of IL-6, TNF-α, and IL-8 were elevated in the bronchoalveolar lavage (BAL) of individuals exposed to Zn oxide particles [13]. Moreover, IL-6 was identified as a sensitive and early systemic biomarker for exposure to metal fumes containing Zn and Cu [17]. Therefore, we focused on IL-6, TNF, and IL-8 secretion of lung epithelial cells, and macrophages exposed to Zn/Cu-containing metal particles. These key inflammatory cytokines were also shown to be regulated in lung epithelial cells and macrophages exposed to a particular matter [20]. The chemokine MIP-1β/CCL4, secreted by macrophages [30], is involved in macrophage activation [31] and chronic inflammation [32]. Therefore, MIP-1β/CCL4 was selected to investigate the cell response to further particle exposure. The presented in vitro study was designed to achieve a comprehensive understanding of the mechanisms underlying the (immuno-)toxicological and inflammatory effects of welding fumes across a wide concentration range in epithelial cells (A549) and monocyte-derived macrophages (THP-1). Moreover, this approach may help to identify potential biomarkers for risk assessment in the context of occupational health surveillance.

## 2. Results

### 2.1. Cytotoxicity

**Figure 1:**
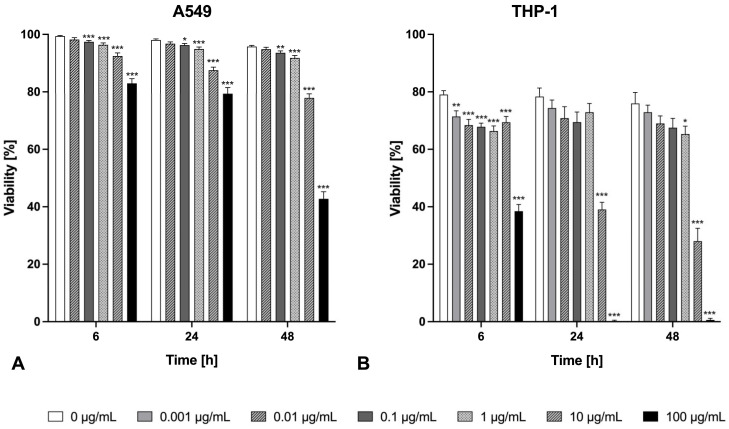
Assessment of cell viability by trypan blue exclusion test of A549 epithelial cells (**A**) and THP-1 macrophages (**B**) after exposure to Zn/Cu-containing welding fume particles. Viability of different particle concentrations (0–100 µg/mL) over the exposure time (6 h, 24 h, and 48 h). Each measuring point shows the mean ± SEM for *n* = 15 (A549) or *n* = 12 (THP-1). (*) *p* < 0.05, (**) *p* < 0.01, (***) *p* < 0.001.

To evaluate the cytotoxic impact of Zn/Cu-containing welding fume exposure on human alveolar lung epithelial A549 cells and THP-1 macrophages, the uptake of trypan blue was determined. The viabilities of both exposed cell lines are presented at different concentrations at various time points (6 h, 24 h, and 48 h) (Figure 1).

The welding fume exposure induced a time- and concentration-dependent cytotoxic effect on human alveolar epithelial cells and macrophages. In direct comparison, the macrophages reacted more sensitively (Figure 1B) than the lung epithelial cells (Figure 1A). The negative controls (0 µg/mL) showed that THP-1 (80.13 ± 1.83%) is less viable at the beginning of exposure than A549 (98.86 ± 0.42%) as a result of the previous macrophage differentiation. Generally, both cell lines already showed a significant decrease in viability at lower particle concentrations (0.001 µg/mL THP-1; 0.1 µg/mL A549). Exposure to 10–100 µg/mL welding fume particle concentrations lead to a viability reduction of 20–56% for A549 cells over the incubation time in comparison to the negative control. Under the same conditions, THP-1 cells showed a viability reduction of 50 to 80% (or higher).

### 2.2. Apoptosis

**Figure 2:**
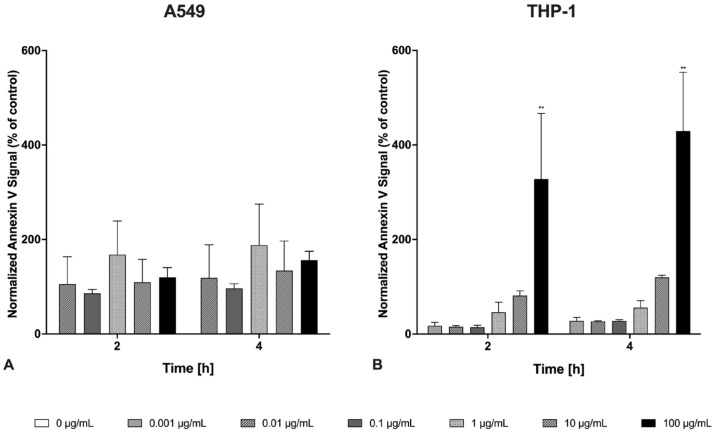
Annexin V staining of A549 epithelial cells (**A**) and THP-1 macrophages (**B**) during exposure to Zn/Cu-containing welding fume particles, evaluated by the Incucyte^®^ live-cell analysis system. The graph shows the annexin V fluorescence signal area (orange) to phase confluence area ratio. Baseline correction was performed by subtracting the non-exposed control values from the exposed cell data at 2 h and 4 h. Data are presented relative to the non-exposed control after 0 h of exposure to varying concentrations of Zn/Cu-containing welding fume particles. Each bar represents the mean ± SEM for *n* = 3. Statistically significant differences between exposed and unexposed cells are indicated with (**) *p* < 0.01.

During apoptosis, phosphatidylserine (PS) translocates to the surface of apoptotic cells, where it can be bound by annexin V. The apoptotic response of cells to welding fume particles was studied using annexin V staining in live cell imaging, measured by normalized orange fluorescence (Figure 2). Fluorescent signals were quantified at different exposure concentrations for 2 h and 4 h exposure times for A549 epithelial cells and THP-1 macrophages. In A549 cells (Figure 2A), the applied exposure doses from 0.01 to 100 μg/mL did not induce any significant increases in annexin V. The highest increase was observed at the exposure dose of 1 μg/mL for both time points; at 2 h, there was a 1.87-fold increase, and at 4 h, the increase was slightly higher. At the exposure dose of 100 μg/mL, there was also a moderate increase over time; at 4 h, there was a 1.55-fold increase approaching statistical significance (*p* = 0.055). In THP-1 macrophages (Figure 2B), the applied exposure doses from 0.001 to 100 μg/mL caused a dose- and time-dependent increase of the annexin V staining. At lower concentrations (0.001–0.1 μg/mL), fluorescence remained near baseline levels (approximately 15–27% of control) across both time points. However, substantial increases were observed at the highest concentration. The 100 μg/mL treatment induced statistically significant elevations of approximately 3.28-fold at 2 h and 4.29-fold at 4 h compared to the control. The time profile showed that the fluorescence intensity in THP-1 macrophages generally increased with extended exposure, with the 4 h values exceeding the corresponding 2 h measurements for equivalent concentrations.

### 2.3. Inflammatory Effects

**Figure 3:**
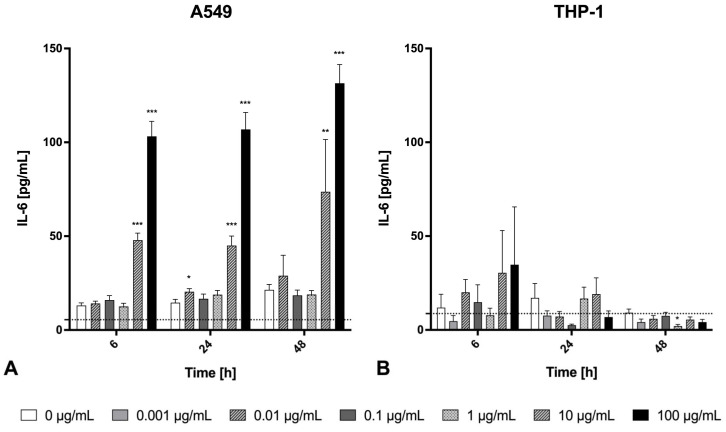
IL-6 release from A549 cells (**A**) or THP-1 macrophages (**B**) after exposure to Zn/Cu-containing welding fume particles at different concentrations (0–100 µg/mL) over time (6 h, 24 h, and 48 h). Each bar shows the mean ± SEM for *n* = 15 (A549) or *n* = 12 (THP-1). Dashed lines show the level of the negative control. Dashed lines show the level of negative control. Statistically significant differences between exposed and unexposed cells (*) *p* < 0.05, (**) *p* < 0.01, (***) *p* < 0.001.

**Figure 4:**
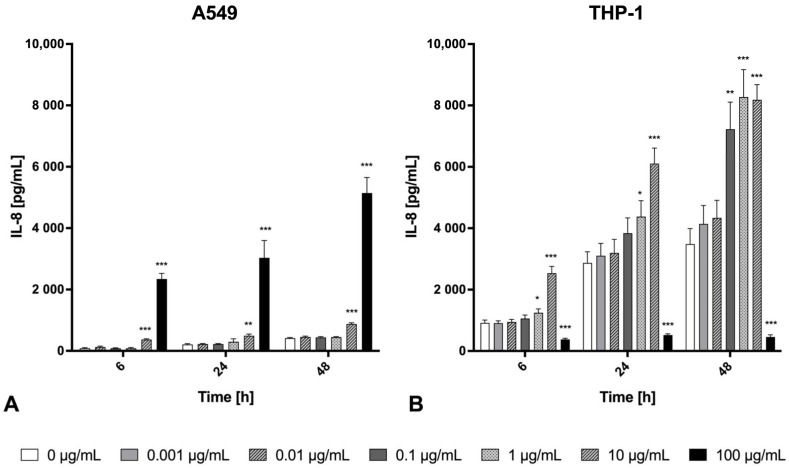
IL-8 release from A549 cells (**A**) or THP-1 macrophages (**B**) after exposure to Zn/Cu-containing welding fume particles at different concentrations (0–100 µg/mL) over time (6 h, 24 h, and 48 h). Each bar shows the mean ± SEM for *n* = 15 (A549) or *n* = 12 (THP-1). Statistically significant differences between exposed and unexposed cells (*) *p* < 0.05, (**) *p* < 0.01, (***) *p* < 0.001.

**Figure 5:**
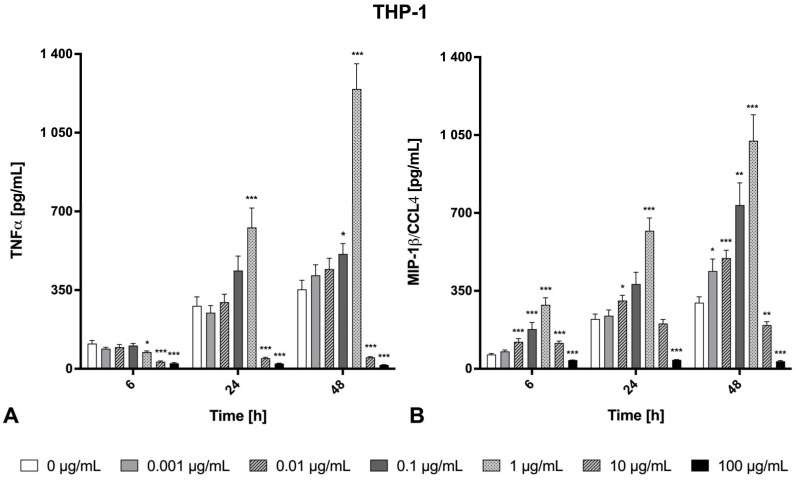
TNF-α (**A**) and MIP-1β/CCL4 (**B**) release from THP-1 macrophages after exposure to Zn/Cu-containing welding fume particles at different concentrations (0–100 µg/mL) over time (6 h, 24 h, and 48 h). Each bar shows the mean ± SEM for *n* = 15 (A549) or *n* = 12 (THP-1). Statistically significant differences between exposed and unexposed cells (*) *p* < 0.05, (**) *p* < 0.01, (***) *p* < 0.001.

**Figure A1:**
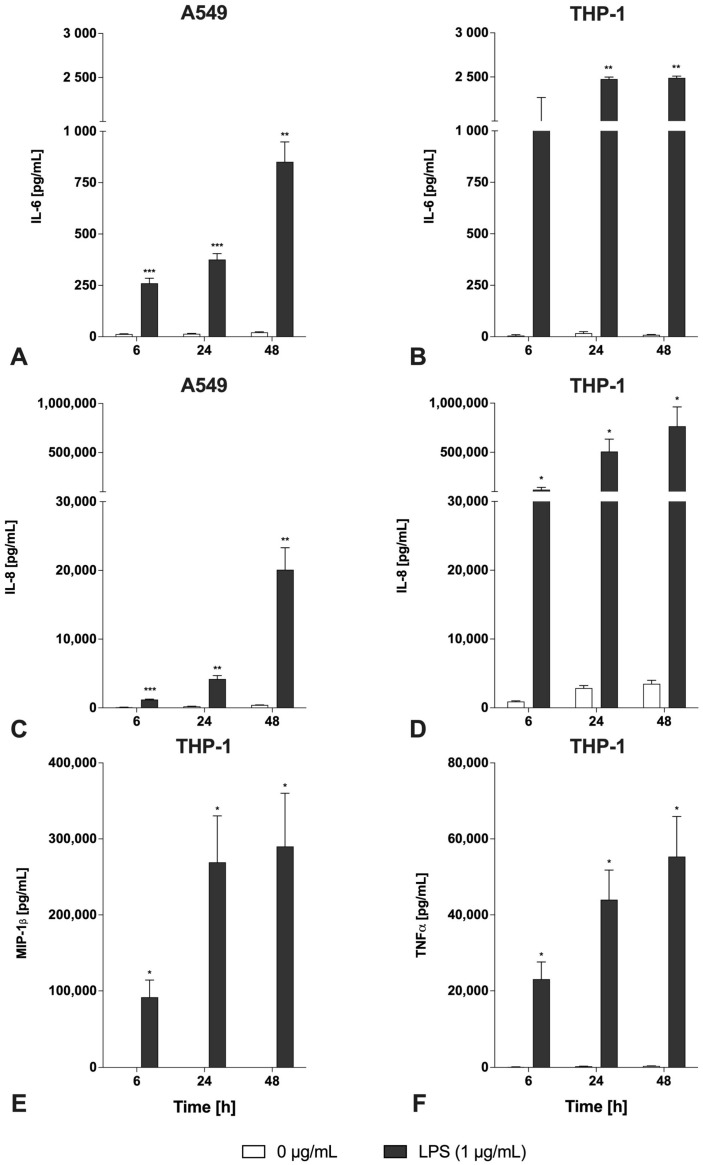
Cytokine release from A549 epithelial cells and THP-1 macrophages after 6 h, 24 h, and 48 h exposure to 1 µg/mL LPS as a positive control for a pro-inflammatory response. Each bar shows the mean ± SEM of at least 3 independent experiments. (**A**) IL-6 of A549. (**B**) IL-6 of THP-1 macrophages. (**C**) IL-8 of A549. (**D**) IL-8 of THP-1 macrophages. (**E**) MIP-1β/CCL4 of THP-1 macrophages. (**F**) TNF-α of THP-1 macrophages. Statistically significant differences between exposed and unexposed cells (*) *p* < 0.05, (**) *p* < 0.01, (***) *p* < 0.001.

The release of the inflammatory cytokines TNF-α and IL-6, as well as the chemokines MIP-1β/CCL4 and IL-8, were determined in the cell supernatant after 0 h, 6 h, 24 h, and 48 h of welding fume exposure (Figure 3, Figure 4 and Figure 5). With respect to physiological function and available data about the secretion spectrum of both cell lines, TNF-α and MIP-1β/CCL4 were only investigated for THP-1 macrophages. In contrast, the secretions of IL-6 and IL-8 were quantified for both cell lines. In general, the inflammatory marker secretion showed a clear cell line-, concentration-, and time-dependent cytokine release. As a positive control for the pro-inflammatory response, we used LPS at a relatively high dose of 1000 ng/mL, which resulted in a significant and time-dependent increase in IL-6 and IL-8 secretion in both A549 epithelial cells and THP-1 macrophages, as well as a significant and time-dependent increase in MIP-1β/CCL4 and TNF-α in THP-1 macrophages (Figure A1).

The production and release of IL-6 is presented in Figure 3. The exposed alveolar epithelial cells (A549) showed a clear concentration- and time-dependent increase in IL-6 secretion compared to the control (Figure 3A). The maximal IL-6 releases in A549 cells were already detected after 6 h at a high particle concentration of 10 µg/mL. In comparison, no significant changes in secreted IL-6 levels were found for exposed THP-1 macrophages compared to the control (Figure 3B). Possible interferences of cytotoxicity could be determined for THP-1 macrophages (but not A549 cells) after treatment with concentrations > 10 µg/mL at 24 h and 48 h, which is made evident by the stagnated or decreased trend in IL-6 concentrations (Figure 3B).

Lung epithelial cells and macrophages showed increased IL-8 secretion in a time- and concentration-dependent manner (Figure 4). The levels of IL-8 secreted by macrophages increased approximately 30-fold after 48 h of exposure to 1 µg/mL particles compared to unexposed control at the same time point, reaching 8300 pg/mL. At the highest particle concentration (100 µg/mL), the IL-8 secretion showed a significant cytotoxicity-based decrease in THP-1 cells. In comparison, the alveolar epithelial A549 cells demonstrated an approximately 1.6–20-fold increase in IL-8 compared to unexposed control at the same time point, with a maximum of 5140 pg/mL (at 100 µg/mL after 48 h). THP-1 macrophages showed increased TNF-α as well as MIP-1β/CCL4 secretion after exposure to Zn/Cu-containing welding fume suspension in a time- and concentration-dependent manner (Figure 5). Regarding the decreased IL-6, TNF-α, and MIP-1β/CCL4 levels at 10 and/or 100 μg/mL fume particle exposure concentration at 24 h and 48 h (Figure 3 and Figure 5), the cytotoxicity values (depicted in Figure 1) will have to be considered. At lower concentrations, THP-1 macrophages secreted high concentrations of TNF-α. At the exposure concentration of 1 µg/mL, the first significant increases of TNF-α could be detected at 24 h and reached a high release of 1244.4 ± 112.2 pg/mL after an incubation period of 48 h. In comparison to TNF-α, the chemokine MIP-1β/CCL4 was significantly released by THP-1 macrophages already at an earlier time point (6 h) and at a lower exposure concentration of 0.01 µg/mL. Furthermore, THP-1 macrophages significantly released MIP-1β/CCL4 at 0.001 µg/mL of the Zn/Cu-containing welding fume suspension after 48 h exposure time.

### 2.4. Genotoxicity

The genotoxicity of Zn/Cu-containing welding fumes was assessed using the umu-test with Salmonella typhimurium TA 1535 [pSK 1002]. The umuC gene is part of the cell’s SOS repair system that counteracts damage to the bacterial genetic material.

The test was carried out both with and without S9 microsomal rat liver extract for the metabolic activation of potential genotoxins. For all exposure concentrations of the test material, the growth factors (GFs) were in the range of 0.83–1.24 (Table 1), and thus, the bacterial growth inhibition was clearly less than 50%, which is the prerequisite for the validity of the test result. The induction ratios (IRs) were from 0.88 to 1.24 (Table 1), indicating that the welding fumes did not induce genotoxicity for any of the welding fume suspension concentrations. Thus, no genotoxic effects of the Zn/Cu-containing welding fume were detected.

### 2.5. Oxidative Stress

**Figure 6:**
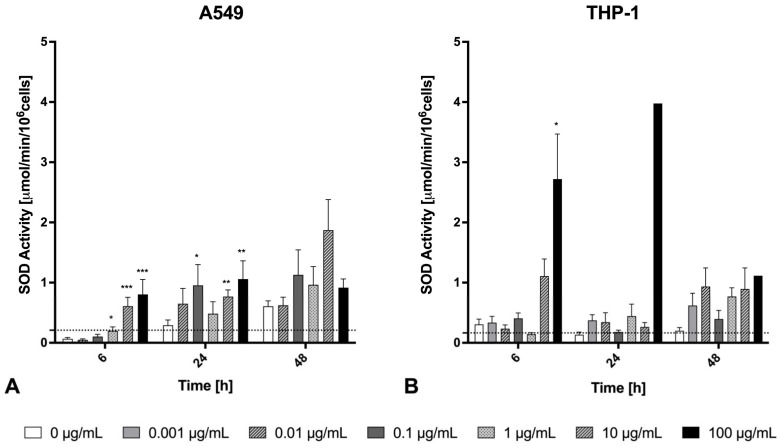
SOD activity in A549 cells (**A**) or THP-1 macrophages (**B**) after exposure to Zn/Cu-containing welding fume particles at various concentrations (0–100 µg/mL) over time (6 h, 24 h, and 48 h). Each bar shows the mean ± SEM for *n* = 4. Dashed lines show the level of the negative control. Statistically significant differences between exposed and unexposed conditions are highlighted as follows: (*) *p* < 0.05, (**) *p* < 0.01, and (***) *p* < 0.001.

The activity of the defense enzyme SOD was measured as an indirect method to analyze whether exposure to welding fumes results in the formation of reactive oxygen species, which could lead to oxidative stress in the cells. In A549 epithelial cells, a significant to highly significant increase in SOD activity was observed following treatment with concentrations ranging from 1 to 100 μg/mL at 6 h. The highest enzyme activity recorded for these cells was approximately 2 μmol/min/10^6^ cells after 48 h of exposure to 10 μg/mL fume particles (Figure 6A). In contrast, THP-1 macrophages exhibited a different response. When exposed to the highest concentration for 6 h, SOD activity was found to be 2-fold higher compared to A549 under the same conditions. The highest enzyme activity recorded for macrophages was about 4 μmol/min/10^6^ cells after 24 h of exposure to 100 μg/mL fume particles (Figure 6B). For both cell lines, the increases in SOD activity were most consistent at the 6 h time point (Figure 6). Regarding the lower SOD activity values at the 100 μg/mL fume particle exposure concentration at 48 h (Figure 6), the cytotoxicity values (depicted in Figure 1) will have to be considered.

## 3. Discussion

Toxic airborne ultrafine metal particles are typically inhaled in the airways and may cause lung inflammation and cytotoxicity. Two essential cell types in the lung are alveolar epithelial cells and alveolar macrophages. Therefore, we tested the toxicological and inflammatory effects of suspensions of the occupationally relevant Zn/Cu-containing welding fume on human A549 alveolar epithelial cells and PMA-differentiated human THP-1 macrophages in vitro in comparable experimental settings. Our study demonstrates differential (time- and concentration-dependent) toxicological and inflammatory effects of Zn/Cu-containing welding fume in both cell types: (i) We found, to the best of our knowledge for the first time, that THP-1 macrophages significantly secreted MIP-1β/CCL4 at an exposure concentration of 0.01 µg/mL after 6 h and 0.001 µg/mL after 48 h, thereby suggesting MIP-1β/CCL4 as a highly sensitive inflammatory biomarker for human macrophages after Cu- and Zn-containing welding fume exposure. (ii) THP-1 macrophage exposure to 1 µg/mL welding fume particles caused significantly increased IL-8 and TNF-α concentrations in the supernatant at 6 and 24 h, respectively. THP-1 macrophage exposure with a 10-fold lower concentration (0.1 µg/mL) caused significantly increased IL-8 and TNF-α concentrations in the supernatant after 48 h. (iii) A549 typically do not secrete TNF-α and MIP-1β/CCL4, and significant increases of IL-6 and IL-8 were determined in A549 supernatants only at high exposure concentrations of 10 µg/mL and particularly 100 µg/mL. (iv) Overall, THP-1 macrophages reacted more sensitively to the Zn/Cu-containing welding fume suspension exposure than A549 cells, as indicated by earlier and/or more pronounced increases of SOD activity, apoptosis rates measured by annexin V staining and cell cytotoxicity percentages. Notably, while A549 cells proliferate during exposure, THP-1 macrophages do not, which may contribute to the observed heightened sensitivity of THP-1 macrophages to Zn/Cu-containing welding fume suspensions. (v) No genotoxic effect of the Zn/Cu-containing welding fume could be detected.

Using relatively high, constant bolus doses of manufactured nanoparticles during the exposure period is common practice in in vitro particle research. Some representative examples are ZnO and CuO particles on lung epithelial cells and macrophages (0.1 to 330 µg/mL) [23], ZnO nanoparticles on macrophages (36 µg/mL) [24], and metal particles containing high levels of Zn and Cu on endothelial cells (0.1 to 50 µg/mL) [33]. In our study, we used a wide concentration range of Zn/Cu exposure doses from 1 ng/mL to 0.1 mg/mL in order to extend the typically tested dose ranges, especially at lower exposure concentrations. In order to relate these in vitro values to real-world occupational conditions, the authors of McCarrick et al. (2022) developed a methodology [34] based on the computational Multiple-Path Particle Dosimetry (MPPD) model (www.ara.com/mppd; accessed on 1 March 2025). The MPPD model is used to simulate the behavior of aerosols in the respiratory system and to calculate nasal, tracheobronchial, and pulmonary deposition of inhaled (nano)particles on the basis of particle properties such as particle diameter and mass concentration, under the consideration of mucociliary and macrophage-mediated clearance. To enable direct comparison with in vitro studies, the authors of McCarrick et al. (2022) converted deposited mass into deposition per surface area, dividing tracheobronchial deposits by 3220 cm^2^ of airway surface area and alveolar deposits by 790,000 cm^2^ of alveolar surface. The general OEL used in the regulation of Zn welding fumes corresponds to 5 mg/m^3^ according to IARC [35], which translates to 0.89 µg/cm^2^ for tracheobronchial deposition (TBD) and 0.017 µg/cm^2^ for alveolar deposition (ALD) after 6 h of exposure.

To compare these values to cytotoxic doses from in vitro cell cultures, this concentration-to-area ratio approach is used for the cell culture Petri dishes, each containing adherent cells in 5 mL of medium on 21 cm^2^ of growth surface. For example, a particle concentration of 0.1 µg/mL means that we have (in 5 mL) a total of 0.5 µg of particles, which, divided by the 21 cm^2^ growth area, results in 0.024 µg/cm^2^. This concentration is 2.7% of 6h-TBD and 1.41-fold that of 6h-ALD. Thus, 0.1 µg/mL (corresponding to 0.024 µg/cm^2^) is less than the 6h-TBD but above the 6h-ALD and close to the 1 week-ALD (Table A1). Based on this methodology, the concentrations of 0.001, 0.01, 0.1, 1, 10, and 100 µg/mL in the cell culture medium applied in the current in vitro study correspond to concentration-to-area ratios of around 0.00024, 0.0024, 0.024, 0.24, 2.4, and 24 µg/cm^2^, respectively. A detailed comparison with exposure classification is shown in Table A1.

When using A549 cells as a model system for lung research, the type of culture medium influences the outcome of toxicity assays. Although it is still common to use DMEM as a culture medium for A549, e.g., [36,37,38], Cooper et al. (2016) demonstrated that long-term cultures of A549 cells in Ham’s F-12K medium for up to 25 days had a transcriptomic profile more similar to primary alveolar type II (ATII) cells compared to A549 cells cultured in DMEM [39]. In addition, A549 cells cultured in DMEM medium showed increased crowding and piling up compared to Ham’s F-12K only after continuous long-term cultures of up to 25 days but not after short-term cultures [39]. Consistent with this, we did not observe any crowding or piling up of A549 cells in DMEM in our short-term cultures of up to 2 days. Future studies on the toxicity of Cu and/or Zn particles in A549 lung epithelial cells might consider long-term cultures in Ham’s F-12K medium to further verify the physiological relevance of previous results (e.g., this study, Lanone et al. (2009), [23] and Karlsson et al. (2008) [40]). Interestingly, cells exposed to selenium in Ham’s F-12K medium for 1–2 days showed an even higher sensitivity to selenite toxicity than cells cultured in DMEM [41].

When interpreting the SOD data, one should consider that SOD activities do not necessarily reflect oxidative stress. One of the main functions of SODs is to convert superoxide radicals to hydrogen peroxide and molecular oxygen [42]. The SOD activity kit used in this study is designed to detect the total catalytic activity of Cu,Zn-SOD (SOD1), Mn-SOD (SOD2), and Cu,Zn-SOD (SOD3). As the names indicate, SOD1 and SOD3 contain Cu and Zn. Considering that the main intracellular SOD is Cu,Zn-SOD, thus having both Cu and Zn as co-factors, a direct effect of Cu/Zn-addition to cells on SOD activity may be suggested. For example, oral Zn supplementation increased both gene expression and catalytic activity of SOD in overweight patients [43]. Therefore, the Zn/Cu concentration-dependent increase in SOD activity in the current study may indicate a higher protection of the cells from oxidative stress at medium Zn/Cu concentrations. In contrast, decreased SOD activity levels could—in view of the cytotoxicity data—indicate increased oxidative stress at higher metal concentrations. In a recent study, high concentrations of CuO nanoparticles (25 µg/mL) have been reported to cause SOD1 misfolding in macrophages, exacerbating oxidative stress and proteasomal dysfunction [28]. In order to further elucidate the oxidative stress status of the Zn/Cu-exposed cells, future studies should incorporate the direct detection of ROS production (for example, via the DCFH assay [44] or other fluorescence-based probes) in order to be able to correlate SOD activity with ROS levels [45]. Furthermore, one should consider that SOD1 does not only act as an antioxidant but also activates nuclear gene transcription [46].

This study found significantly elevated chemokine MIP-1β/CCL4 secretion by THP-1 macrophages after Zn/Cu-containing welding fume exposure. The higher exposure concentration of 10 µg/mL and especially 100 µg/mL showed inflammatory effects in A549 cells but caused stronger cytotoxic effects in THP-1 macrophages after 24 h to 48 h, confirming a higher sensitivity of THP-1 macrophages towards Zn/Cu-exposure in comparison to A549 cells. The secretion of MIP-1β/CCL4 by THP-1 macrophages was also observed in response to a low concentration of 0.001 µg/mL Zn/Cu-containing welding fume suspension. In contrast, TNF-α secretion by THP-1 macrophages was only induced at a concentration approximately 100-fold higher at 0.1 µg/mL. This finding underlines that MIP-1β/CCL4 is a highly sensitive biomarker for macrophages, as its original name indicates. The study did not identify the lowest effective concentration of Zn/Cu-containing welding fume required to induce MIP-1β/CCL4 secretion in THP-1 cells, as no lower concentrations than 0.001 µg/mL were tested. However, it is unlikely that clearly lower concentrations than 0.001 µg/mL could cause MIP-1β/CCL4 release, as exposure to 0.001 µg/mL metal particles caused detectable significantly elevated levels of secreted MIP-1β/CCL4 only after 48 h.

From a regulatory perspective, this study demonstrated the sensitivity of this human-relevant in vitro biomarker MIP-1β/CCL4, suggesting its further validation for integration into standardized macrophage-based New Approach Method (NAM) assays for nanomaterial risk assessment. Future applications of MIP-1β/CCL4 as macrophage-specific sensitive biomarkers could help safety assessment for emerging materials by enabling parallel screening of inflammatory potential and mechanistic toxicity across human cell systems, complementing existing NAMs focused on acute cytotoxicity. Integrating such sensitive in vitro models into regulatory decision-making would accelerate evidence-based updates to exposure guidelines while reducing animal testing burdens. This is not only useful for high-production volume metals such as Cu and Zn, but the proven ability to detect biological activity at environmentally relevant concentrations may establish this approach as a fundamental methodology for next-generation risk assessment frameworks that balance industrial innovation with worker health protection.

MIP-1β/CCL4 affects immune and non-immune cells by binding them to its main receptor, CCR5. MIP-1β/CCL4 is particularly known as one of the major HIV-suppressive factors [31], and increased systemic MIP-1β/CCL4 levels have been suggested as potential biomarkers of severe COVID-19 [47]. MIP-1β/CCL4 secretion by THP-1 monocytes was detected after 24 h of exposure to nickel sulfate (100 µg/mL) and cobalt sulfate (130 µg/mL) [48]. While these metal concentrations are relatively high, the specificity of MIP-1β/CCL4 induction in response to metals suggests its utility as a biomarker. Notably, MIP-1β/CCL4 production is mechanistically linked to canonical inflammatory pathways, such as TLR4-MyD88 signaling and downstream NF-κB/AP-1 activation, which are shared between lipid-mediated metabolic inflammation and metal-induced stress responses [49,50]. For instance, induction of MIP-1β/CCL4 occurs when THP-1 cells are co-stimulated with TNF-α and palmitate, a process dependent on clathrin-mediated endocytosis and MAPK/NF-κB signaling [49]. This suggests that MIP-1β/CCL4 production may result from direct metal stimulation and secondary inflammatory signaling cascades. Moreover, MIP-1β/CCL4’s functional role in recruiting monocytes to sites of inflammation underscores its potential biological relevance as a marker of macrophage activation during metal exposure [50,51]. Significantly increased MIP-1β/CCL4, found in bronchoalveolar lavage fluid (BALF) of smokers affected by chronic bronchitis compared to smoking or non-smoking healthy control groups, showed a moderate negative correlation between MIP-1β/CCL4 levels and forced expiratory volume in one-second values (*p* = −0.64, *p* = 0.035) of chronic bronchitis [32]. This suggests that MIP-1β/CCL4 might be a lung inflammation biomarker and exerts pro-inflammatory and immune-modulatory effects. Current biomonitoring approaches under investigation for metal workers exposed to welding fumes or metal particles primarily focus on measuring urinary or blood concentrations of toxic metals (e.g., chromium, manganese, nickel) [52] and oxidative stress biomarkers like 8-oxo-7,8-dihydroguanosine (8-oxoGuo) and 3-nitrotyrosine, which reflect RNA/DNA damage from reactive oxygen species generated by metal exposure [53] and standard inflammatory markers [54,55,56]. Given its role as a chemokine produced during macrophage-mediated inflammatory responses, MIP-1β/CCL4 could complement existing biomarkers by assessing chronic inflammation risks [57,58]. This approach could help to recognize inflammatory pathologies in high-risk occupations at an early stage.

Controlled short-term inhalation of Zn- and Cu-containing welding fumes increases the systemic acute-phase mediator IL-6 and the acute-phase proteins CRP and SAA [16,17,18,59]. CRP is a risk marker for future cardiovascular diseases (CVDs) [9], and IL-6 and SAA are regarded as risk markers and even mediators for CVD [9,60,61,62]. Furthermore, epidemiological studies show that long-term welders have a higher risk of CVD [5,63]. Recent proteomic analyses of welders’ sera reveal dose-dependent changes in CD84 and FGF23, proteins implicated in vascular inflammation and cardiac remodeling, even at exposures below current occupational limits (1–5 mg/m^3^) [64,65]. Of note, hypertensive patients with high MIP-1β/CCL4 quartiles face elevated cerebrocardiovascular risks [66]. Elevated systemic levels of MIP-1β/CCL4 correlate with an increased risk of stroke and cardiovascular events [67]. Experimental models further show that MIP-1β/CCL4 inhibition attenuates atherosclerotic plaque development by reducing macrophage infiltration and stabilizing vulnerable lesions, highlighting its therapeutic potential [67]. Overall, future studies should investigate whether elevated MIP-1β/CCL4 levels can be detected in serum, airway secretions, and/or exhaled breath condensate of metal workers, including welders. In this context, it is important to consider that in real-world scenarios, the metal workers being tested should not have any infections that could cause a similar increase in biomarkers.

IL-6 activates macrophages and T cells and is a potent acute-phase mediator, which causes the liver to release acute phase proteins like CRP and SAA [68]. This study found that A549 alveolar epithelial cells secrete high levels of IL-6 after exposure to Zn/Cu-containing welding fumes. This possibly provides a potential link to the previous findings of elevated systemic IL-6 (and CRP and SAA) levels in healthy individuals after controlled Zn/Cu-containing welding fume exposure [17]. The chemokine IL-8 recruits leucocytes, particularly neutrophils, to inflammatory sites. In our study, we found its release by both cell types, A549 and THP-1 macrophages, with the levels secreted by the macrophages being higher than those secreted by lung epithelial cells. This may be due to the macrophages’ ability to internalize foreign particles. The fact that THP-1 cells were overall more sensitive than A549 cells in the current study is consistent with previous reports: A549 cells are known to be more resistant to exposure to nanomaterials [40]. In addition, THP-1 macrophages reacted more sensitively than A549 cells to CuO and ZnO particles for both single or mixed metal particles [23].

## 4. Materials and Methods

### 4.1. Welding Fume Generation

The welding process described in the study involves metal inert gas (MIG) soldering, a technique commonly used in vehicle manufacturing for joining galvanized steel. This process utilizes a low-alloy copper wire as the filler material, which is applied to hot-dip galvanized steel substrates. The base metal used is DX51D + Z275 (1.0226), classified under EN 10346 standards [69], while the filler material is CuSi3Mn (Bercoweld S3, Bedra, Germany), composed of 96% copper, 1% manganese, and 3% silicon, conforming to ISO 24373 specifications [70] with a diameter of 1.2 mm. Argon serves as the shielding gas during the soldering process, which operates in pulsed arc mode to ensure precise control of heat input and material deposition. The fumes generated during this process consist of ultrafine particles with a modal diameter of approximately 122 ± 1.46 nm. Detailed information regarding the welding process and materials can be found in research conducted by Hartmann et al. in 2014 [56].

### 4.2. Welding Fume Characterization via Inductively Coupled Plasma Mass Spectrometry (ICP-MS)

The composition of the welding fume was analyzed using inductively coupled plasma mass spectrometry (ICP-MS). A mixture of 50 mg of welding fume, 1 mL Rhodium-ICP-Standard solution, 7 mL hydrogen peroxide, and 5 mL aqua regia (75% (*v*/*v*) HCl, 25% (*v*/*v*) HNO_3_) was digested for 1 h in the microwave. For measurement, the digested welding fume was diluted 1:10 with aqua bidest. Standards for elements aluminum, arsenic, barium, beryllium, boron, cadmium, calcium, cobalt, copper, chromium, iron, lead, lithium, magnesium, manganese, molybdenum, nickel, phosphorus, rubidium, selenium, strontium, tellurium, thallium, tin, vanadium, and zinc were used for quantification. The main elemental metal components of the welding fume are 8% copper oxide and 70.2% zinc oxide. Additionally, the welding fume contains a remaining part that shows traces of iron, manganese, calcium, phosphorus, and others in varying amounts. A proportion of the weight of the remaining welding fume particles may be due to the presence of oxygen from metal oxides. We refer to it as Zn- and Cu-containing welding fume or Zn/Cu-containing welding fume because it mainly consists of large amounts of zinc and significant portions of copper.

### 4.3. Preparation of the Welding Fume Extracts and Exposure Solutions

The welding fume was collected as a dark grey powder and subsequently extracted in an assay medium using sonication for 30 min to produce a stock exposure solution of 1000 µg/mL. Following the sonication process, a 1:10 dilution series was prepared to yield five (A549) or six (THP-1) particle extracts, each at final concentrations of 100, 10, 1, 0.1, 0.01, and 0.001 µg/mL (only THP-1). The preparation and dilution of the extract solutions were conducted under strict sterile and standardized conditions. The resulting welding fume exposure solutions were aliquoted and stored at −20 °C until usage.

### 4.4. Controlled Experimental In Vitro Exposure to Welding Fumes Containing Zinc and Copper on A549 Lung Epithelial Cells and THP-1 Macrophages

#### 4.4.1. Cell Culture and Culture Conditions

The A549 cell line represents a human alveolar basal epithelial cell line derived from a type II-like lung carcinoma. It was obtained from the cell line service (CLS, Eppelheim, Germany). Unless specified, the cell culture reagents were obtained from Gibco (Thermo Fisher Scientific Inc., Waltham, MA, USA), ensuring consistent results. The cell line was cultured as a monolayer in Dulbecco’s Modified Eagle Medium (DMEM) cell culture medium containing GlutaMAX, 10% (*v*/*v*) FBS (Invitrogen, Darmstadt, Germany), 1% (*v*/*v*) penicillin (100 IU/mL) and streptomycin (100 µg/mL). The monocytic cell line THP-1 has been isolated from the blood of an acute monocytic leukemia patient (male). The cell line was cultured as suspension cells in Roswell Park Memorial Institute (RPMI 1640) cell culture medium containing GlutaMAX, 10% (*v*/*v*) FBS (Invitrogen, Darmstadt, Germany), 1% (*v*/*v*) penicillin (100 IU/mL), and streptomycin (100 µg/mL). The monocytes were differentiated into macrophages by culturing the cells in the above-mentioned culture medium spiked with 200 nM phorbol 12-myristate 13-acetate (PMA; Sigma-Aldrich GmbH, Steinheim, Germany) and 0.1% (*v*/*v*) dimethyl sulfoxide (DMSO; Sigma-Aldrich GmbH, Steinheim, Germany). All cell lines were grown in sterile plastic flasks (175 cm^2^, Greiner Bio-One, Frickenhausen, Germany) in a humidified 5% CO_2_ atmosphere at 37 °C.

#### 4.4.2. Experimental In Vitro Exposure Procedure and Sample Preparation

Cells were seeded at a density of 5 × 10^5^ cells (A549) and 2.4 × 10^6^ (THP-1) in 5 mL respective cell culture medium per cell culture dish (60 mm; Greiner Bio-One, Frickenhausen, Germany) and incubated at 37 °C in a humidified atmosphere containing 5% CO_2_ for 48 h. After this time, the culture or differentiation medium was removed to eliminate non-adherent cells and washed thrice with prewarmed phosphate-buffered saline (PBS). Only vital adherent and differentiated (THP-1) cells were incubated with assay medium (cell culture medium with reduced FBS to 1% (*v*/*v*), without phenol red indicator and with 200 mM L-glutamine instead of GlutaMAX) containing 0.001 (only THP-1), 0.01, 0.1, 1, 10 and 100 µg/mL welding fume for 0 h, 6 h, 24 h, and 48 h. The lowest concentration of 0.001 µg/mL was only used for THP-1 cells, given the internal preliminary study results concerning the high sensitivity of this cell line to the particles. Non-exposed cells were incubated with assay media only and used as a negative control. An assay medium containing 1 µg/mL Lipopolysaccharides (LPS) from Escherichia coli 0111:B4 (Sigma-Aldrich GmbH, Steinheim, Germany) was used as a positive control. After every incubation period (0 h, 6 h, 24 h, and 48 h), 2 mL of cell culture supernatants were collected to isolate the extracellular protein fraction, and an additional 20 µL were collected for viability testing. The 2 mL of supernatant was centrifuged (100× *g*, 5 min), transferred to aliquots, and frozen immediately at −80 °C to determine later on the cytokine and chemokine amount secreted by the cells.

#### 4.4.3. Cytotoxicity by Assessing the Cell Viability

The cytotoxicity of Zn/Cu-containing welding fume was evaluated by staining the cells with trypan blue to distinguish between viable and nonviable cells. After the experimental exposure, 20 µL of detached cells in solution were mixed with 20 µL trypan blue solution and transferred to a Neubauer counting chamber. Viable cells appear light, while nonviable cells appear dark blue under the microscope due to trypan blue permeating the cell membrane and dyeing the cytosol dark blue. The number of viable and nonviable cells was determined for each sample, and the percentage ratio was then calculated to assess cell viability.

#### 4.4.4. Cytokines and Chemokines Detection Cell Culture Supernatant

The levels of secreted TNF-α (K151QWD), IL-8 (K151RAD), and MIP-1β/CCL4 (K151NRD) in the cell culture supernatants were measured using the electrochemiluminescence immunoassays (V-Plex) from Meso Scale Discovery (Meso Scale Diagnostic, Rockville, MD, USA). The levels of secreted IL-6 were analyzed using an enzyme-linked immunosorbent assay (ELISA) BD OptEIA™ Human IL-6 Set (555220; BD Biosciences, Franklin Lakes, NJ, USA). All assays were conducted following the instructions provided by the manufacturers.

#### 4.4.5. Genotoxicity Assessment Due to Exposure to Welding Fumes Containing Zinc and Copper

The reporter gene assay umu-test is a genotoxicity test for detecting chemical mutagens and carcinogens [71]. The test system is based on the induction by alkylating agents of SOS functions coupled to a reporter gene in the umuC lacZ fusion plasmid. For the investigation, the test strain *Salmonella typhimurium* TA1535/pSK1002 was used for the investigation. The genotoxicity activities were determined by measuring the β-galactosidase activity. The test performance was realized according to DIN 38415-3:1996-12 [72]. Deviating from ISO 13829 [73], the samples were extracted in a 100% assay medium without antibiotics and introduced into the test system. The extent of induction of the SOS repair system was indicated by β-galactosidase units, which result in the induction rate (IR). According to the standard (ISO 13829), a sample was supposed to be genotoxic if this induction rate exceeds 1.5. For the welding fume sample, the first dilution with an induction rate below 1.5 and a growth factor exceeding 0.5 is presented (Lowest ineffective dilution: DLI-value).

#### 4.4.6. Annexin V Detection Using the Sartorius Incucyte^®^ SX5 Live-Cell Analysis System for Live-Cell Imaging

The THP-1 cells were seeded in a 96-well plate (Sarstedt, Nümbrecht, Germany) at a density of 2.5 × 10^4^ cells per well and were then incubated for 48 h in RPMI 1640 with GlutaMAX (Gibco, Waltham, MA, USA) medium containing 10% (*v*/*v*) fetal bovine serum (FBS; PAN-Biotech, Aidenbach, Germany), 1% (*v*/*v*) penicillin and streptomycin (Gibco, Waltham, MA, USA), and 200 nM phorbol 12-myristate-13-acetate (PMA; Sigma-Aldrich, Saint Louis, MO, USA) to allow for differentiation and attachment at 37 °C, 5% CO_2_, and 95% humidity. Furthermore, A549 cells were seeded in a 96-well plate (Sarstedt, Nümbrecht, Germany) and were grown in DMEM with GlutaMAX (Gibco, Waltham, MA, USA) containing 10% (*v*/*v*) FBS (PAN-Biotech, Aidenbach, Germany) and 1% (*v*/*v*) penicillin and streptomycin (Gibco, Waltham, MA, USA). These cells were then incubated for 48 h to allow attachment and the formation of a cell monolayer. After the 48 h incubation period, the cells were washed three times with sterile PBS (Gibco, Waltham, MA, USA). Subsequently, each well was spiked with 5 µM of annexin V inert dye (4759; Sartorius, Goettingen, Germany). The control samples remained unexposed, while the treated cells were exposed to varying concentrations (0.001, 0.1, 1, 10, and 100 µg/mL) of Zn/Cu-containing welding fumes for 72 h (37 °C, 5% CO_2_, 95% humidity) in the Sartorius Incucyte^®^ SX5 Live-Cell analysis system for live-cell imaging. The concentrations of the exposure treatments corresponded to those indicated in the text and the figures. Images from the scan interval were analyzed using the Sartorius IC Incucyte^®^ software 2023A. The generated data was further processed and visualized using GraphPad Prism 10.0.0 (GraphPad Software, Boston, MA, USA).

#### 4.4.7. Determination of Oxidative Stress via Superoxide Dismutase (SOD)

Commercial colorimetric assays determined superoxide dismutase after cell disruption in the cell extract. Superoxide dismutase was performed using the Superoxide Dismutase Assay Kit by Sigma-Aldrich (19160; Sigma Aldrich, Steinheim, Germany) and was performed according to the manufacturer’s instructions.

### 4.5. Statistical Analysis

Statistical analyses were performed using GraphPad Prism 10.0.0 (GraphPad Software, Boston, MA, USA) and MedCalc 23.0.5 for Windows (MedCalc Software 23.0.5, Ostende, Belgium). Results are presented as mean values ± SEM. For each incubation time point (2 h, 4 h, 6 h, 24 h, and/or 48 h), data from cell cultures exposed to welding fumes were compared to the non-exposed control cultures. Two statistical tests were used: the Student’s *t*-test for normally distributed data and the Mann–Whitney U test for non-parametric data. Both tests were two-sided tests to account for potential differences in either direction. Statistical significance was defined as *p* ≤ 0.05.

## 5. Conclusions

The increasing industrial use of Zn and Cu across sectors ranging from welding technologies to nanotechnology applications highlights the importance of assessing the safety of occupational exposure to metal fumes, as exposure to those particles is associated with potential health risks. The toxicological analyses in this in vitro study indicated that exposure to Zn/Cu-containing welding fumes caused cell-specific cytotoxicity, apoptosis, increased SOD activity, and inflammatory cytokine responses. A distinct secretion of MIP-1β/CCL4 by THP-1 macrophages occurred at low Zn/Cu-containing welding fume suspension concentrations, down to 0.001 µg/mL, at a concentration when none of the other tested cytokine was observed. This suggests that MIP-1β/CCL4 could be a highly sensitive biomarker for exposure to metal-containing particles and that it may be useful to validate its integration into standardized macrophage-based NAM assays for sensitive safety assessment of emerging (nanoscale) materials. The chemokine MIP-1β/CCL4, along with IL-8 and TNF-α secreted by THP-1 cells as well as IL-6 and IL-8 produced by A549 cells at higher concentrations, contributes to local lung inflammation and potential systemic effects and may increase cardiovascular risk in chronically exposed individuals. The release of IL-6 by A549 cells may stimulate acute phase protein production in the liver, explaining previously observed elevated systemic inflammatory markers in welders. The differential cellular responses highlight the importance of studying multiple cell types when assessing occupational and environmental exposures.

## Figures and Tables

**Table 1:** Results of genotoxicity testing using the umu-test for different concentrations of Zn/Cu-containing welding fume particles (0–100 µg/mL). Each value represents the mean ± SEM of one independent experiment in triplicate. The following terminology denotes specific assay parameters: Dilution Factor for Induction (DLi value), Induction Ratio (IR), and Growth Factor (GF).

	Without Metabolic Activation				With Metabolic Activation			
	D_Li_	IR	GF	Genotoxicity	D_Li_	IR	GF	Genotoxicity
Blank (Assay medium)	100	1.11 ± 0.23	0.99 ± 0.1	-	100	1.03 ± 0.11	0.97 ± 0.06	-
Welding fume [µg/mL]								
0.001	100	1.06 ± 0.08	1.01 ± 0.11	-	100	0.88 ± 0.03	1.09 ± 0.05	-
0.01	100	1.06 ± 0.08	1.24 ± 0.11	-	100	1.06 ± 0.06	1.10 ± 0.06	-
0.1	100	1.0 ± 0.08	1.12 ± 0.13	-	100	1.0 ± 0.05	1.16 ± 0.03	-
1.0	100	1.07 ± 0.10	1.07 ± 0.11	-	100	0.89 ± 0.17	1.13 ± 0.04	-
10	100	1.24 ± 0.05	0.95 ± 0.11	-	100	1.07 ± 0.05	0.89 ± 0.08	-
100	100	1.05 ± 0.06	1.15 ± 0.15	-	100	1.24 ± 0.12	0.83 ± 0.11	-

## Data Availability

The data that support the findings of this study are available from the corresponding author upon reasonable request.

## Appendix B

**Table A1:** Based on McCarrick et al. (2022) [34], the table highlights the differences between in vitro concentrations (0.00024–24 µg/cm^2^) and human inhalation data for the occupational exposure level (OEL) of 5 mg/m^3^ welding fume, corresponding to 0.89 µg/cm^2^ for tracheobronchial deposition (TBD) and 0.017 µg/cm^2^ for alveolar deposition (ALD) after 6 h of exposure (short-term exposure). One week of this exposure was calculated to 0.102 µg/cm^2^ TBD and 0.083 µg/cm^2^ ALD, and one year to 1.15 µg/cm^2^ TBD and 2.85 µg/cm^2^ ALD (long-term exposure). This enables a comparison of in vitro and realistic occupational exposure data.

		In Vitro vs. Human Inhalation		In Vitro vs. Human Inhalation		Exposure Classification
**In Vitro**	**In Vitro**	**1-Week TBD**	**1-Week ALD**	**1-Year TBD**	**1-Year ALD**	
**[µg/mL]**	**[µg/cm^2^]**	**(0.102 µg/cm^2^)**	**(0.083 µg/cm^2^)**	**(1.15 µg/cm^2^)**	**(2.85 µg/cm^2^)**	
-	-	0.102	0.083	1.15	2.85	OEL 6 h as baseline (5 mg/m^3^)
0.001	0.00024	0.24%	0.29%	0.021%	0.0084%	Realistic short-term exposure
0.01	0.0024	2.4%	2.9%	0.21%	0.084%	Realistic short-term exposure
0.1	0.024	23.5%	28.9%	2.1%	0.84%	Upper limit of short-term exposure
1	0.24	2.35-fold	2.89-fold	20.9%	8.4%	Most representative for long-term exposure
10	2.4	23.5-fold	28.9-fold	2.09-fold	16%	Exceeds occupational thresholds
100	24	235-fold	289-fold	20.9-fold	8.4-fold	Exceeds occupational thresholds
